# Network Medicine for Alzheimer’s Disease and Traditional Chinese Medicine

**DOI:** 10.3390/molecules23051143

**Published:** 2018-05-11

**Authors:** Juliet T. Jarrell, Li Gao, David S. Cohen, Xudong Huang

**Affiliations:** 1 Neurochemistry Laboratory, Department of Psychiatry, Massachusetts General Hospital and Harvard Medical School, Charlestown, MA 02129, USA; jjarrell@smith.edu (J.T.J.); DCOHEN17@mgh.harvard.edu (D.S.C.); 2 Modern Research Center for Traditional Chinese Medicine, Shanxi University, Taiyuan 030006, China; gaoli87@sxu.edu.cn

**Keywords:** Alzheimer’s disease, network medicine, inflammation, network and system pharmacology, traditional Chinese medicine

## Abstract

Alzheimer’s Disease (AD) is a neurodegenerative condition that currently has no known cure. The principles of the expanding field of network medicine (NM) have recently been applied to AD research. The main principle of NM proposes that diseases are much more complicated than one mutation in one gene, and incorporate different genes, connections between genes, and pathways that may include multiple diseases to create full scale disease networks. AD research findings as a result of the application of NM principles have suggested that functional network connectivity, myelination, myeloid cells, and genes and pathways may play an integral role in AD progression, and may be integral to the search for a cure. Different aspects of the AD pathology could be potential targets for drug therapy to slow down or stop the disease from advancing, but more research is needed to reach definitive conclusions. Additionally, the holistic approaches of network pharmacology in traditional Chinese medicine (TCM) research may be viable options for the AD treatment, and may lead to an effective cure for AD in the future.

## 1. Introduction

Over 5 million people in the United States currently have Alzheimer’s disease (AD), and it is predicted that this number will reach 16 million by 2050. AD is a senile dementia, which is a medical condition that involves memory loss and cognitive impairment. Around 60 to 80 percent of dementia patients have AD, making it therefore the most common type of dementia. Most AD patients are 65 years old or older, however, people of any age can be diagnosed with AD. AD has become a pandemic as the population continues to age, and at the present time there is no known cure and prevention measure for AD.

The principles of NM have recently been applied to AD research and have led to many important findings about the disease. Network medicine is a new and continually developing field, centered around the idea that a disease does not just affect a single gene via a mutation, but rather that many genes and components of a much larger pathway could be involved in the progression of a disease. Furthermore, studying similarities between multiple diseases can reveal these gene and pathway components. The disease module is an important principle of NM; genes that are involved in the same diseases interact more and express more similar transcription factors than genes that are not involved in the same diseases, and these similar genes constitute a disease module [1]. In addition, the human diseasome is important to the field; the diseasome is a huge map of information about how diseases manifest based on their relevant disease genes [1]. The diseasome was built by combining the human disease network, which is a map of how diseases can be linked if they share a gene mutation or more than one gene mutation that corresponds to both diseases, and the disease gene network, which is a map of linkages between two genes if they play a role in the same disease [1]. Because of the diseasome, diseases can now be studied in association with their disease genes, and more can be learned about how these genes build the disease pathway; it is no longer believed that diseases are the product of one gene. 

Furthermore, in 2015, it was discovered that disease proteins that are involved in 226 diseases come together in the same area of the interactome, which is the sum of interactions in a cell, and this collusion creates a higher probability that a disease module will form [2]. Diseases are not only based on connections, as the diseasome revealed, but are also based on the interactions that happen in a certain area. Most recently, in 2017, a software called RWRB was used to map five networks made up of genes that are similar to one another, and these networks were called Integrated Gene Similarity Networks (ISGNs). The ISGNs also include phenotype similarities and gene-phenotype relationships [3]. Models of disease networks based on gene similarities have been built based on this RWRB software. These findings and other similar findings in the field of NM have fueled AD research specifically in the areas of functional network connectivity, particular components of the disease network including myelination and myeloid cells, and genes and pathways in order to decipher how the disease manifests. Combinations of network pharmacology and TCM approaches as possible avenues for treatment and a cure have also been investigated.

## 2. Functional Network Connectivity

As AD spreads, connectivity of neurons in the brain and the function of the brain both decrease, and the brain essentially becomes dysfunctional. There are a number of studies that show that AD has an adverse effect on normal functional network connectivity [4,5,6], and one study even suggests that functional connectivity in the default mode network could be a potential AD biomarker [7]. The cause of this decrease in connectivity and function is not known, and thus there is no way to stop or delay the disease progression at this time. Many studies have applied the principles of NM to investigate the stages of connectivity and function as they relate to the disease, in an attempt to learn more about how the disease progresses. 

In one study, functional connectivity and cortical thickness of the brain were investigated in 98 subjects, both healthy patients and patients afflicted with AD [8]. For each person, brain cortical thickness data and functional connectivity of the cortical network data was gathered using the functional Magnetic Resonance Imaging (fMRI) method. There were significant differences in cortical thickness and functional connectivity for healthy patients and AD patients [8]. Therefore, functional connectivity and cortical thickness could be important markers of the AD progression, but more research on these topics is required to reach a definitive conclusion. A previous study also found that functional connectivity decreased in patients with mild cognitive impairment (MCI) as compared to patients with normal cognitive activity in regards to the bilateral medial frontal, parahippocampal, precuneus, middle temporal, right superior temporal, and left angular gyri regions of the brain [9]. The cognitive function and connectivity decrease in different areas of the brain as a result of AD should continue to be explored.

Similarly, in another study, the fMRI technique was once again used, this time to create network maps of the brain that involve connectivity and brain function. The study proposes that using a minimum spanning tree for high-order functional networks is the most effective strategy because this minimum spanning tree takes into account constant changes in brain connectivity [10]. In addition, the study suggests the use of an optimization framework that displays the most crucial pieces of the functional connectivity network [10]. If this method involving a minimum spanning tree and an optimization framework is used, then more can be learned about the spread and progression of AD on a network level while factoring in the constant changes of functional connectivity. A similar study proposed that using orthogonal minimal spanning trees (OMSTs) and optimization would be most effective for modeling brain networks [11]. However, mapping networks in the brain is extremely complicated, and these studies do not discuss potential problems and gaps in knowledge.

The effect of different molecules on functional network connectivity has also been investigated. One study looked at the effects of tau, which is a protein associated with neurodegenerative disease such as AD, on functional network connectivity in P301L mice [12]. EEG oscillations were used to investigate the relationship between the prefrontal cortex and the hippocampus. These oscillations revealed phase-amplitude cross frequency coupling (PAC) between gamma and theta oscillations, which occurs during normal brain function and is important for this normal brain function. Studying the oscillations also revealed how auditory cues create oscillations and utilize the mismatch negativity (MMN) principle to look at synthesis of information in the brain. It was discovered that the relationship between the hippocampus and the prefrontal cortex became weaker as early as the second week of the study, and in addition, PAC gamma and theta oscillations became weaker in the same time frame [12]. Also, tau introduction utilized the MMN principle, because it produced an altered response of oscillations; this alteration suggests that memory and recall were affected by the tau introduction [12]. In summation, tau introduction weakens theta and gamma oscillations and network connectivity.

Likewise, another study tested whether or not tau introduction in the entorhinal cortex could disturb local field potentials in surrounding areas in rats, and the results proved that tau creates abnormal circuit activity during learning [13]. Moreover, a recent study reported that tau networks play a role in AD [14]. Altogether, these findings about tau suggest that it may be an important factor in AD development, but it is not the only component of AD pathology, and thus research about tau introduction needs to be combined with study about the other aspects of neurodegeneration to produce an accurate model of the disease.

Recently, a novel principle about preservation of functional network connectivity in regard to AD was discovered. In the past, neuron excitement and hyperactivity have been observed during the AD manifestation in the cerebral cortex and the hippocampus, and one study found that greater connectivity and subsequent network failure create the disease [15]. A simulation of the AD progression in another recent study was produced to look at how the neurons were affected; six different methods to either excite or hinder the neurons were then attempted in the network simulation. It was discovered that exciting the neurons conserved neuron connections and the network system as a whole most effectively [16]. Based on this principle, excitement of the neurons could slow down or stop the AD progression in the human brain. Because exciting the neurons seems as though it could create more damage in patients with AD, because their brain activity is already decreasing as the disease progresses, more research about the concept of exciting the neurons to preserve the network should be undertaken, and future findings should be compared to this study.

## 3. Particular Components of the Network

### 3.1. Myelination

As the networks and pathways of AD have been investigated, attempts have been made to find out what components of these networks and pathways are important to the spread of the disease. Recent research has shown that AD affects myelination and myeloid cells in the brain in different ways. Because of these findings, myelination and myeloid cells could become targets for AD drug therapy. 

A few studies have investigated myelination and oligodendrocytes in the context of AD. Oligodendrocytes create myelin, which is a substance that surrounds the axon of some cells in the nervous system. Both of these components are critical for brain function, and the purpose of one study was to investigate how these components purportedly break down in the progression of AD [17]. Oligodendrocyte gene networks were created based on transcriptomic, proteomic, and genomic data collected from post-mortem brains affected by AD and data from gene expression in mice that expressed signs of oligodendrocyte and myelin degradation. The oligodendrocyte gene networks that were created consisted of many genes that are known to be involved with AD, and the mice that were analyzed showed the same signs of oligodendrocyte and myelin breakdown as the human brains afflicted with AD [17]. Furthermore, a previous study identified the Bin1 gene in oligodendrocytes as an important focus area for late-onset AD [18], and an even older study discovered that components in the brain which create oligodendrocytes are sensitive to amyloid plaques, which is a feature of AD [19].

Another study explored myelin presence solely in mice; this study observed atypical myelin progression in the hippocampus of APP/PS1 mice that were two months old [20]. Previously, changes were observed in the oligodendrocytes and myelin of mice after amyloid plaques and tau were introduced [21] and the aforementioned findings were expanded on; In this recent study, it was discovered that oligodendrocyte development is atypical in these mice [20], suggesting that the mice might be a valid model for AD in humans, because the progression of AD in humans is also not a normal part of aging. Neuregulin-1 Type III, a protein involved in oligodendrocyte and myelin development, was found to be upregulated in the hippocampus of the mice [20]. In addition, neuregulin-1 Type III can be cut by active-caspase-6 in the cytoplasm [20]. These findings about specific components of myelin and oligodendrocyte pathways can be applied to human AD research because myelin and oligodendrocyte appear to be important to AD progression. Myelin and oligodendrocyte gene networks should be further analyzed in order to learn more about how these components are integrated into the AD model and in order to decide if these components could possibly be AD drug targets.

One gene study made further discoveries about myelination and its connection to AD; it identified that certain genes involved in AD are incorporated in myelination and a few other significant processes [22]. In this study, previous data and research concerning different brain regions of various AD patients were taken into account, as well as 1596 samples of brain areas from 1103 AD patients. Once the samples were collected, algorithms were then used to decipher gene co-expression and networks in each area of the brain that was studied. It was discovered that genes for synaptic processes, mitochondrial processes, the immune system, and myelination are involved in AD [22]. As a next step, scientists need to decipher exactly how each of these processes may come together to create AD pathology.

### 3.2. Myeloid Cells

Myeloid cells, which respond to inflammation in the brain, have been a popular topic for AD research in addition to myelination. In a recent study, 14,406 genomes affected by AD were researched and 25,849 healthy patients were also investigated, and a gene network that involves myeloid cells and is specifically related to AD was discovered [23]. This study utilized the information in a previous study, which identified eleven regions of interest for late-onset AD, including myeloid cells [24]. In this study, the transcription factor PU.1 was discovered, and this is a protein that controls gene expression; PU.1 may, as a result of its function, control the entire myeloid network. SPI1, which is a gene that codes for the transcription factor PU.1, therefore may also control this myeloid cell network.

To test if SPI1 does control the network, mouse microglia was explored, and microglia are a main type of myeloid cell. When SPI1 was suppressed in mice microglia, there was not as much phagocytosis in the cells, but if SPI1 was overexpressed, there was increased phagocytosis [23]. The presence of phagocytosis in mouse cells does not necessarily mean that SPI1 is a master regulator of the AD network, but it certainly presents an argument for further studies in this area. Additionally, genes that control the age at which a person contracts AD are present in myeloid cells, and that a low level of SPI1 and, subsequently, a low level of PU.1, means that a person will contract AD at an older age [23]. These findings may help doctors decipher who is susceptible to AD and who is less susceptible. Unfortunately, PU.1 is involved with many microglial cells, thus it would not be a good drug target specifically, and more research needs to be conducted on this myeloid network in order to isolate another component of the gene network that would be easier to target without destroying vast amounts of microglial cells. If another component is isolated, this discovery may spark the creation of a potential AD drug that could be effective.

Another study also discovered a connection between AD and myeloid cells. DNA from 85,000 patients was analyzed in this study, and three new gene variants for AD were isolated through a genome wide association study (GWAS); these genes code for three proteins which are housed in microglia cells [25]. Based on these findings about microglia and myeloid cells, determinations should be made about if AD drugs can target microglia cells, and if injury response pathway should be a target for AD treatment.

## 4. Genes and Pathways

Because NM is based on the principle that diseases manifest from a whole set of genes and pathways and not just from one single mutation, then according to this principle, the study of genes and pathways associated with AD could be vital to the search for an effective cure. In one study, there was an attempt to understand more about pathways and genes in senescence accelerated mouse-prone 8 (SAMP8) mice, because these mice are used as a model for AD research [26]. To conduct this study, genes expressed in the hippocampus and cerebral cortex of 2 month, 6 month, and 12 month old SAMP8 mice were used as compared to SAMR1 mice of the same age. A sub-network of genes in the brain was constructed using PPI, MetaCore, and the co-expression network. It was discovered that transmission and apoptosis were not regulated properly in these mice, and 10 genes were abnormally expressed: RAF1, MAPT, PTGS2, CDKN2A, CAMK2A, NTRK2, AGER, ADRBK1, MCM3AP, and STUB1 [26]. 10 microRNAs were also discovered: miR-20a, miR-17, miR-34a, miR-155, miR-18a, miR-22, miR-26a, miR-101, miR-106b, and miR-125b that could regulate the sub-network [26]. Based on these findings, more is now known about the genetic networks of SAMP8 mice and their neurological functions. SAMP8 mice should continue to be used for AD research, because they display signs of abnormal gene expression networks in the brain and neurological deficits.

In another genetically based study, the category of certain genes associated with AD and the relationships between these genes were investigated [27]. 430 genes that are involved in AD as identified from 830 scientific works and publications about AD genes were used for this study. Once the genes associated with AD were identified, the particulars of those genes were researched, and then the crosstalk as a result of the pathways and relationships between the genes was investigated. Another study that was published a few months later used a similar approach for Parkinson’s Disease (PD), suggesting that the network and pathway based approach may be a viable one for studying neurodegenerative diseases [28].

In the Alzheimer’s study, it was also discovered that genes in the Alzgset (Alzheimer’s disease related genes gene set) are possibly involved in lipid and lipoprotein function, immune system function, metabolic processes, drug responses, and neurological development [27]. The genes are also involved in visual learning, sleep, axon activity, behavior, and memory. In addition, three main pathway types that the genes were associated with were isolated: neuronal-related or xenobiotic/drug metabolism related pathways, cell growth/survival and neuroendocrine signaling pathways, and immune response pathways [27]. The functions of the three pathways are all linked to one another, which means that AD probably affects all three components and is controlled by all three components. In the previous section of this review concerning myelination and myeloid cells, other research about the immune system’s involvement in AD was also discussed, and thus the immune system is somehow clearly involved in AD. Now that these pathways have been isolated, further research needs to be done about how they may be specifically involved in the AD progression.

A similar study investigated whether a model for AD based on pathways that incorporate gene expression data instead of simply the gene expression data itself would be more productive [29]. The study used two different data sets of gene expression that already existed, and accumulated new gene expression data from blood samples. There was no observable difference in gene and pathway models, but only one data collection approach was used in this particular experiment. More pathways should be tested and more techniques should be employed to decipher pathways, because a pathway level approach makes sense for AD since the disease affects full scale network connectivity. A previous study indicated a connection between gene expression in blood and the AD onset [30], thus findings at the pathway level may exist as well. Based on these findings about genes and pathways, some genes that are present in AD patients have been isolated, but further research about new genes and how these genes relate to one another to form pathways still needs to be conducted. Information about genes and pathways relating to AD is a constantly evolving topic, because there are a huge amount of genes that may be associated with AD, and an even larger amount of connections between them.

## 5. Network and Pathway Modeling

Based on the principle of NM which states that diseases are multi-faceted, new research about AD should take into account the network of the disease as a whole, and not just specific pieces, in order to observe the full spread of the illness. One study used GWAS on the hippocampus in two sets of subjects in the Alzheimer’s Disease Neuroimaging Initiative (ADNI) [31]. These GWAS were then applied to a network-wide association study (NetWAS) that was tissue-specific, and this network-wide association study attempted to make connections in the GWAS to create a hippocampal network of pathways that may be involved in AD. The process of this study shows that construction of network models may provide important findings for AD, because these networks help to model what is happening in the progression of the disease on many levels. However, no specific findings about the connections in the GWAS were reported, therefore, not much concrete evidence can be gathered from this study. A more recent study also identified a tissue-specific network approach for research about amygdala imaging [32], thus this multifaceted network technique may be viable, but more research is needed to prove its validity.

The use of predicted pathways models is common so that more can be learned about the AD network. In one particular study, protein-protein interaction (PPI) networks were combined with gene expression information to produce a model of the signaling pathway of AD [33]. Since inflammation is thought to be an important factor in the development of AD, for the model of the signaling pathway, NF-kB, which creates inflammation, started the pathway. A pathway that travels from NF-kB gene to an AD-associated protein- amyloid precursor protein (APP), was then reconstructed. As a result of the signaling pathway model, six harmful genes were isolated that are a part of the pathway, and therefore these genes could be integral to AD [33]. However, since it is not known if NF-kB definitely plays a role in AD, or even if it starts this pathway, this model is all theoretical, and does not definitively reach any conclusions. In a previous study, it was discovered that NF-kB may play a possible signaling role in AD [34], but more research about NF-kB is needed in order to find out more specific information.

One study suggests the use of CNN (convolutional neural network) models to help detect AD and MCI [35]. The 16-layered VGGnet CNN for AD patients, MCI patients, and healthy controls was changed via a dataset from the Alzheimer’s Disease Neuroimaging Initiative (ADNI). Based on this DemNet method, accurate detection of AD, MCI, or healthy brain function in patients was made 91.85% of the time [35]. CNN models should continue to be experimented with, as 91.85% accuracy in detection is extremely efficient. 

## 6. Traditional Chinese Medicine (TCM) and Network Pharmacology

Network pharmacology has recently been applied to traditional Chinese medicine (TCM) research in AD. TCM therapy and network pharmacology both operate on the philosophy that a more holistic approach to illness is preferable. TCM has been in practice for thousands of years, and TCM doctors today continue to use a variety of herbal medicines and practices that involve the mind and body, such as tai chi and acupuncture, to restore the entire human system. Similarly, scientists in the field of network pharmacology are currently attempting to treat complex diseases like AD by developing drugs that acts on multiple targets of the disease.

Recent research has revealed that TCM may help to treat patients afflicted with AD, and may help in the discovery of a cure. For example, one study looked at how gingko biloba affects patients dying of dementia [36]. The 3777 patients, made up of people who consumed ginkgo biloba, people who consumed other drugs, and people who did not consume any drugs, were evaluated every two years. It was discovered that patients have a lower risk of dying if they consume ginkgo biloba [36]. Similarly, another group of scientists studied the effects of ginkgo biloba on symptoms of dementia as compared to the effects of placebo in 1628 patients over a period of twenty two to twenty four weeks, and found that, according to the caregivers, patient symptoms improved significantly more for those who were given the ginkgo biloba as opposed to those who were given the placebo [37]. In addition, TCM anti-aging herbs may be effective for AD patients, as AD is not a normal part of aging. One study investigated the effects of Liuwei Dihuang decoction (LW), a TCM herb, on SAMP8 mice; SAMP8 mice have been used as a model for late onset and age-related AD, and a study previously mentioned in this review [26], looked at the genes and pathways relevant to the study of AD in these SAMP8 mice. The results of the TCM LW study showed that LW had an anti-aging effect on these mice, and had favorable effects on their locomotor activity, object recognition, spatial learning, memory, and avoidance impairment [38].

One study proposes that a combination of conventional therapy drugs and Chinese herbal therapy could be the solution to AD. 344 patients diagnosed with AD were treated either with conventional therapy drugs or a combination of conventional therapy drugs and Chinese herbal therapy [39]. The study was conducted over a period of 24 months, and at the beginning of the study, most patients had mild or moderate AD. After 18 months, based on results of the Mini-Mental State Examination (MMSE), patients treated with a combination of conventional therapy drugs and Chinese herbal therapy scored 1.76 points higher than patients treated with only conventional therapy drugs, and after 24 months, patients treated with the combination scored 2.52 points higher than patients treated with only conventional therapy [39]. This study presents an argument for a multifaceted approach to AD treatment, and a combination of conventional single target therapy and TCM herbal therapy.

### 6.1. The Effects of Particular TCM Herbs and Mixtures 

Many studies have investigated the mechanisms of particular TCM herbs and mixtures on AD using NM principles. One study attempted to discover more about the pathways and functions of TCM herbal therapies that have been proven to help AD patients [40]. A combination of text-mining, drug-likeness filtering, prediction of targets, and network analysis was used to investigate the herbal medicines based on a network pharmacology approach. Text-mining of PubMed showed that 10 herbs have a significant relationship with AD, and 1016 compounds for 10 herbs remained after drug-likeness filtering [40]. The compound-target and target-pathway networks were constructed to investigate how these herbs work [40]. More should be learned about TCM herbs from a NM perspective, to discover if they would be a viable treatment option for targeting the complicated AD network. 

In addition, the unknown effects of Danggui-shaoyao-san (DSS), a widely used herbal formula in TCM that been used to treat neurodegenerative diseases such as AD, have been investigated [41]. Biological networks were constructed that looked at interactions between compounds, targets, and neurodegenerative diseases based on the targets of the herbal formula, the ingredients of the formula, and information about how the diseases progress. Based on these networks, more about how DSS works in the human body can be understood. These type of discoveries about the mechanism of TCM herbs can aid the development of better treatment strategies and may lead to more information about the AD network.

One study investigated the network effects of compounds in Erzhi Wan, a TCM remedy, on AD [42]. A network pathway model for Erzhi Wan using the Cytoscape software was established; it was found that the components quercetin, geraniol, β-sitosterol, nerol, and eriodyctiol that exist in Erzhi Wan may be compounds associated with AD, and that Erzhi Wan works with three signaling pathways, Wnt, MAPK, and PI3K-Akt-MTOR [42]. Erzhi Wan and its effects on the full-scale network of AD should continue to be studied.

Moreover, a study examined the effects of ginsenoside Re (G-Re), a component of ginseng, which is a root used in TCM, on mice injected with beta amyloid, which is a common feature of AD [43]. The mice were injected with G-Re for 30 days. The mice’s cognitive function was evaluated based on a maze test, and pathological changes in their brain tissue were evaluated by immunohistochemistry. 10 biomarkers were isolated in these mice, and levels of tryptophan, hexadecasphinganine, phytosphingosine, and multiple lysophosphatidylcholines were lower in the AD mice as opposed to the control, while phenylalanine levels were higher in AD mice as opposed to the control [43]. Furthermore, G-Re was involved in all of these pathways. Because G-Re is involved in so many pathways, G-Re should be further investigated in the development of network pharmacology-based drugs.

### 6.2. Suppression of Inflammation by TCM

Based on a network approach, it has been discovered that certain TCM herbs can help to suppress inflammation, and neuroinflammation is a common facet of AD. In one study, the mechanism of action (MOA) for a couple of TCM herbs was investigated; these herbs have been clinically tested and evaluated to have favorable but yet unknown effects on AD [44]. By looking at the ingredients of the herbs and the proteins that the herbs target, a network framework was constructed to investigate the effects of the herbs upon AD progression as a whole. The results showed that these herbs bind to similar targets as conventional therapy drugs, and also bind to targets that have been implicated in other diseases, such as cancer and diabetes; therefore, AD and these diseases may have an intricate relationship [44]. In addition, as statistical analysis showed, the TCM herbs targeted Ca^2+^ equilibrium networks that stop cell proliferation and inflammation [44]. 

A third study also discovered a connection between inflammation and TCM in the NF-kB signaling pathway, which may be an important part of the AD network. Shenfu injection (SFI) is currently used to treat coronary heart diseases and stroke, but this study showed that it contains compounds that help to curb inflammation, because these compounds inhibit the activity of NF-kB [45]. A study mentioned previously in this paper [33], modeled a network pathway regulated by NF-kB that could be important to AD, because neuroinflammation has been strongly associated with AD pathology, and another study, also mentioned previously in this paper [34], showed that NF-kB may play an important role in AD signaling pathways. The effects of Huperzine A (HupA) which is an alkaloid derived from the Chinese herb Huperzia serrata, on the NF-kB pathway have even been investigated. It was discovered that HupA downregulates Rela (p65) expression, and p65 is one of the most important factors in the NF-kB pathway [46]. Therefore, HupA could be used to treat AD because of its regulatory role in the NF-kB pathway. NF-kB is clearly a crucial part of the AD network, and may possibly become a drug target of AD.

HupA seems to have more than one favorable effect on AD manifestation. One study showed that HupA reduced levels of beta amyloid, stopped the formation of amyloid plaques, and hyperphosphorylated tau in the cortex and hippocampus of mice implanted with AD genes [47]; Tau, beta amyloid, and amyloid plaques are common neuropathological features of AD. Furthermore, when these mice were fed a high iron diet, HupA was no longer able to produce these desired effects. HupA decreases the amount of iron in the brain and stops the expression of iron receptors and the uptake of iron in neurons [47]. Based on these findings, HupA may be able to target multiple aspects of the AD network by decreasing iron levels in the brain and curbing neuroinflammation. However, one Cochrane review looked at the effects of HupA on patients with vascular dementia, and found that HupA did not have any significant effect on cognitive function, measured by the MMSE [48]. More research about the effectiveness of HupA for patients with neurological disorders should be conducted.

### 6.3. Inhibition of AD Network Components

Many studies have discovered that TCM herbal drugs inhibit components that play a role in the AD network. For example, Apolipoprotein E4 (Apo E4) is a genetic risk factor for late onset AD, and one study investigated three main compounds in TCM, solapalmitine, isodesacetyluvaricin, and budmunchiamine L5, that, based on the results of virtual drug screening, have the ability to inhibit Apo E4 [49]. The results showed that budmunchiamine L5 is the most favorable compound for AD treatment, because it has a good absorption and blood-brain barrier (BBB) penetration rate, and is less toxic than the other two alternatives [49]. Additionally, the inhibition of β-site APP cleaving enzyme 1 (BACE1) was studied [50]. The BACE1 is thought to possibly cause AD, because it participates in an important step in the production of amyloid beta. TCM components that could inhibit BACE1 were investigated, and the effects of these components were evaluated using a molecular dynamics simulation [50]. It was discovered that triptofordin B1 can bind to BACE1 and inhibit it effectively, and is less toxic than pyrimidine analogue, which has similar binding abilities [50]. The inhibitory effects of triptofordin B1 and budmunchiamine L5 on the AD network should be further investigated. 

Moreover, the inhibition of acetylcholinesterase (AChE) is a common form of treatment for early stage AD. In one study, the AChE inhibitory effects of methanol, dichloromethane and aqueous crude extracts from 80 plants that are used in TCM were investigated [51]. Extracts of *Berberis bealei* (formerly known as *Mahonia bealei*), *Coptis chinensis* and *Phellodendron chinense* inhibit AchE. In addition, combinations of the individual alkaloids berberine, coptisine, and palmatine cooperatively inhibited AChE [51]. Another enzyme associated with AD is human histone deacetylase 2 (HDAC2), and one study utilized the largest TCM database to investigate compounds that could inhibit HDAC2 [52]. Multiple linear regressions, support vector machines, and the Bayes network toolbox were used to predict how the most significant TCM compounds from the database would inhibit HDAC2. Based on results of these predictions, the compounds Bontl ferulate, monomethylcurcumin, and ningposides C inhibit HDAC2 most effectively [52]. These extracts and compounds should be investigated to see if they have other favorable effects on the AD network.

## 7. Conclusions

Functional network connectivity, myelination, myeloid cells, genes and pathways associated with AD, TCM, and network pharmacology may be important pieces of the puzzle that is still baffling: the cure for AD. Different components of the AD network have been isolated, different pathways associated with the disease have been modeled, and the effects of TCM herbal therapy and network pharmacology approaches have been investigated, but not enough is known about how the AD progression, effective AD cure, and AD onset delay. Further research about the effects of TCM and network pharmacology treatments, the AD pathways and network, the progression of the disease, and the different components of the disease must be conducted, and new approaches to discover more about this complex disease must be adopted. Because NM is such a new and continually evolving field, it has to be more fully developed so that more concrete conclusions from studying AD and other diseases can be made based on its principles and applications. 

## Abbreviations

The following abbreviations are used in this manuscript:

AD	Alzheimer’s Disease
NM	Network Medicine
TCM	Traditional Chinese Medicine
ISGNs	Integrated Gene Similarity Networks
fMRI	Functional Magnetic Resonance Imaging
MCI	Mild Cognitive Impairment
OSMTs	Orthogonal Minimal Spanning Trees
PAC	Phase-Amplitude Cross Frequency Coupling
MMN	Mismatch Negativity
GWAS	Genome-Wide Association Study
SAMP8	Senescence Accelerated Mouse-Prone 8
PPI	Protein-Protein Interaction
PD	Parkinson’s Disease
ADNI	Alzheimer’s Disease Neuroimaging Initiative
NetWAS	Network-Wide Association Study
APP	Amyloid Precursor Protein
CNN	Convolutional Neural Network
LW	Liuwei Dihuang Decoction
MMSE	Mini-Mental State Examination
DSS	Danggui-Shaoyao-San
G-Re	Ginsenoside Re
MOA	Mechanism of Action
SFI	Shenfu Injection
HupA	Huperzine A
Apo E4	Apolipoprotein E4
BBB	Blood-Brain Barrier
BACE1	β-site APP Cleaving Enzyme 1
AChE	Acetylcholinesterase
HDAC2	Human Histone Deacetylase 2
